# Improved efficiency of doubled haploid generation in hexaploid triticale by *in vitro* chromosome doubling

**DOI:** 10.1186/1471-2229-12-109

**Published:** 2012-07-18

**Authors:** Tobias Würschum, Matthew R Tucker, Jochen C Reif, Hans Peter Maurer

**Affiliations:** 1 State Plant Breeding Institute, University of Hohenheim, Stuttgart 70593, Germany; 2 ARC Centre of Excellence for Plant Cell Walls, University of Adelaide, Waite Campus, Adelaide, Urrbrae SA 5064, Australia

## Abstract

**Background:**

Doubled haploid production is a key technology in triticale research and breeding. A critical component of this method depends on chromosome doubling, which is traditionally achieved by *in vivo* treatment of seedlings with colchicine.

**Results:**

In this study we investigated the applicability of an *in vitro* approach for chromosome doubling based on microspore culture. Our results show a pronounced increase in the proportion of doubled haploid triticale plants compared to the spontaneous doubling rate, but also compared to the doubling obtained by the standard *in vivo* approach. In addition, the frequency of plants surviving from culture medium to maturity is also much higher for the *in vitro* approach. Colchicine concentrations of 1 mM for 24 h or 0.3 mM applied for 48 or 72 h during the first hours of microspore culture performed best.

**Conclusions:**

Our results suggest that for triticale, *in vitro* chromosome doubling is a promising alternative to the *in vivo* approach.

## Background

Doubled haploid (DH) technology is a valuable tool in modern breeding programs since it allows for the production of completely homozygous lines within a few months and dramatically reduces the time required to establish new cultivars [1]. In addition, many genomic approaches such as association or QTL mapping benefit greatly from the use of DH populations [2-4]. DH plants are routinely generated in crops such as barley [5]. However, due to high costs and low efficiency, DH production is yet to be established in applied breeding programs for other cereals such as triticale. Of the different methods available for DH production, microspore embryogenesis (androgenesis) shows the greatest potential due to the abundance of microspores per spike and consequently the higher frequency of DH output as compared to other approaches such as wide crosses. Factors limiting the application of microspore culture at a commercial level include the rate of embryogenesis and regeneration, the frequency of albinism among regenerants and the frequency of chromosome doubling required to obtain fertile DH plants [6]. In barley, the rate of spontaneous chromosome doubling is high and ranges from 70-90 % [6]. By contrast, the rate of spontaneous doubled haploids reported for triticale ranges from 6-38 % [7-9], which similar to most crops is too low and variable to omit a specific step for induced chromosome doubling. The most common doubling agent is colchicine which is traditionally applied *in vivo*[10]. This application of colchicine at the seedling stage, however, has several disadvantages: large amounts of colchicine solution are required which imparts high costs and increased exposure to this toxic substance, a high rate of mortality of seedlings owing to the treatment, and production of mixoploid or chimeric plants which results in low seed set and consequently the requirement of additional seed multiplication before field testing [11].

The application of colchicine during early stages of androgenesis could alleviate some of these problems [6] and has been applied to different cereals including wheat [11,12], durum wheat [13] and rice [14]. In wheat, the incorporation of colchicine during the first hours of anther or microspore culture improved the doubling rate [11,12]. The application of doubling agents, however, not only increases the percentage of chromosome doubling, but also affects the whole androgenetic process. The optimum concentration and time of application for chromosome doubling may have negative effects on embryogenesis and regeneration rate, as well as on the percentage of green plants [6]. Thus, *in vitro* chromosome doubling must be investigated and optimized in light of the entire androgenetic process.

In triticale, *in vitro* application of colchicine has been applied in anther culture approaches [7] but not for microspore culture which is emerging as the method of choice. The objective of this work was to investigate the suitability of *in vitro* chromosome doubling during microspore culture for efficient doubled haploid production in triticale. More specifically, the aim was to determine the optimum concentration and incubation period of colchicine for *in vitro* chromosome doubling while considering the androgenetic response as a whole.

## Results

**Figure 1:**
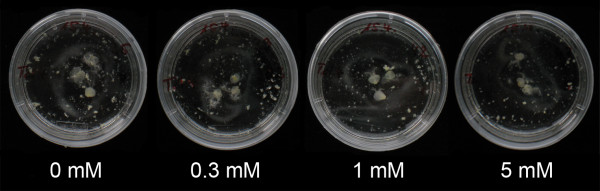
**Effect of colchicine on embryo formation.** Effect of different colchicine concentrations applied for 72 h on embryo formation 4 weeks after microspore isolation.

Visual scoring of Petri dishes containing microspores in embryo induction revealed no obvious effect of the colchicine incubation on the number of regenerated embryos 4 weeks after microspore isolation (Figure 1). In addition, the embryos were of similar high quality and almost no calli were formed.

The regeneration of plants from the transferred embryos averaged 42.7% for the negative controls without colchicine (Table 1) and similar results were obtained for most combinations of colchicine concentration and incubation duration. Lower values were found for the 5 mM concentration at 48 h and 72 h and for 1 mM at 72 h. No clear trend could be observed relating to the percentage of green plants. Whereas colchicine increased the percentage of green plants for the 24 h and 72 h periods, it decreased green plant formation for the 48 h colchicine treatments. In comparison, the *in vivo* approach showed a higher regeneration rate (58.8%).

**Table 1:** **Effect of*in vitro*versus*in vivo*colchicine treatment**

**Colchicine treatment**	**Transferred embryos**	**Regeneration rate**	**Green plants [%]**	**Survival rate [%]**
24 h - 0 mM	330	42.4 a	54.3 a	85.5 a
24 h - 0.3 mM	240	39.2 a	61.7 ab	81.0 ab
24 h - 1 mM	240	41.7 a	72.0 ab	83.3 ab
24 h - 5 mM	180	41.7 a	65.3 ab	71.4 ab
48 h - 0 mM	210	43.3 a	75.8 b	62.3 bc
48 h - 0.3 mM	240	47.1 a	59.3 ab	82.1 ab
48 h - 1 mM	240	47.1 a	69.0 ab	43.6 cd
48 h - 5 mM	120	24.2 b	41.4 a	50.0 ab
72 h - 0 mM	210	42.4 a	48.3 a	60.5 b
72 h - 0.3 mM	240	43.8 a	69.5 ab	68.5 ab
72 h - 1 mM	240	33.3 a	62.5 ab	64.0 ab
72 h - 5 mM	270	26.3 b	71.8 ab	62.7 ab
*in vivo*	835	58.8 c	70.9 b	44.0 d

Survival rate refers to the rate of plants that survived the transfer from culture medium to soil and grew to maturity. Means of columns followed by different letters indicate significant differences at the α = 0.05 level.

The survival rate for plants derived from colchicine-treated seedlings was 44% i.e. approximately half of the green plantlets that were transferred from culture medium could be grown to maturity (Table 1). The remainder died, predominantly within 4 weeks of the colchicine treatment and subsequent transfer to soil. Compared to this *in vivo* chromosome doubling treatment, the survival rate was generally higher for the *in vitro* colchicine approach with an average survival rate of 67.9%. No negative effect was observed for the different colchicine concentrations as compared to the respective controls (Table 1).

**Figure 2:**
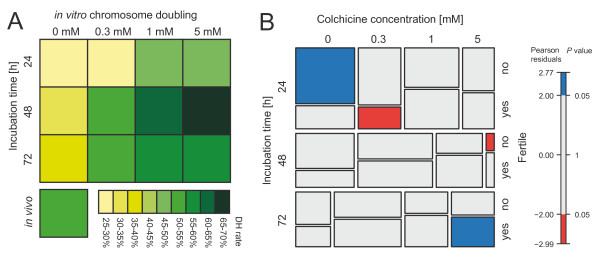
***In vitro*chromosome doubling.(A)** Heatplot for the rate of fertile doubled haploid plants among regenerated green plants obtained by the tested *in vitro* methods and by the conventional *in vivo* approach. **(B)** Mosaic plot visualizing the contingency table. The area of each tile is proportional to the corresponding cell entry and the colors reflect the residuals (fit or lack of fit) of the loglinear model.

The rate of fertile doubled haploid plants from the colchicine treatment at the seedling stage was 52.3% (Table 2), while the percentage of fertile plants in the negative controls, which is equivalent to the spontaneous doubling rate, averaged 32.1%. There was a clear effect of the *in vitro* colchicine treatment on the proportion of fertile plants (Table 2, Figure 2). The highest values, ranging from 56.2% to 66.7%, were obtained for 1 mM and 5 mM concentrations and incubation times of 48 h and 72 h. These values roughly doubled that of the spontaneous doubling rate and were even higher than that obtained by the *in vivo* procedure. Consistent with this, we observed significant variance components of colchicine concentration and incubation time for the proportion of fertile plants (Additional file 1: Table S1). The plants produced mainly fertile tillers as compared to the plants from the *in vivo* approach for which, on average, half of the tillers were sterile (Table 2). There appeared to be no effect on the number of seeds per fertile tiller.

**Table 2:** Effect of the chromosome doubling procedure on plant fertility

**Colchicine treatment**	**Plants**	**Fertile plants [%]**	**Fertile plants per 100 embryos**	**Mean sterile tillers**^**1**^	**Mean fertile tillers**^**2**^	**Mean seeds per fertile tiller**^**3**^
24 h - 0 mM	65	29.2 a	5.8 ab	0.0 a	5.6 cdef	29.1 bcde
24 h - 0.3 mM	47	27.7 a	5.4 ab	0.4 a	6.9 abcd	33.8 a
24 h - 1 mM	60	50.0 ab	12.5 b	0.5 a	4.5 fg	24.2 e
24 h - 5 mM	35	45.7 ab	8.9 ab	0.3 a	4.9 efg	24.1 cde
48 h - 0 mM	43	32.6 a	6.7 ab	0.7 a	7.7 ab	19.8 bcde
48 h - 0.3 mM	55	50.9 ab	11.7 b	0.9 a	7.0 abc	22.4 bcde
48 h - 1 mM	34	61.8 b	8.8 ab	0.5 a	5.7 defg	33.7 abcd
48 h - 5 mM	6	66.7 ab	3.3 a	0.3 a	7.0 a	26.6 ab
72 h - 0 mM	26	38.5 a	4.8 ab	0.9 a	6.3 abcde	32.0 ab
72 h - 0.3 mM	50	52.0 ab	10.8 ab	0.5 a	5.0 defg	31.3 bcde
72 h - 1 mM	32	59.4 ab	7.9 ab	0.9 b	6.5 abcde	27.4 abc
72 h - 5 mM	32	56.2 ab	6.7 ab	0.2 a	6.3 bcde	36.7 a
*in vivo*	153	52.3 ab	9.6 ab	2.9 b	3.7 g	31.0 de

^1^ mean number of sterile and ^2^ fertile tillers per seed-setting plant, and ^3^ mean number of seeds per fertile or partially fertile tiller. Means of columns followed by different letters indicate significant differences at the α = 0.05 level.

## Discussion

For scientists as well as for commercial breeders, the establishment of a routinely applicable doubled haploid (DH) protocol is of significant interest. For triticale, microspore culture has been shown to provide a high frequency of DH plants, and thus is becoming the method of choice for DH production [8,9]. Key issues remain, however, that prevent the widespread application of this method. A major limitation relates to the low frequency of spontaneous DH plants produced in triticale, which necessitates chemically-induced doubling of the chromosomes to obtain fertile plants. In this study we compared the traditionally employed *in vivo* approach to those where colchicine is applied *in vitro* during the first stage of microspore culture.

The *in vitro* application of colchicine has been reported to induce positive as well as negative effects on the whole androgenetic pathway, influencing the events of embryogenesis, regeneration rate and the percentage of green plants [15]. In contrast to Zhou et al. [16], who found an increased frequency of embryogenesis in *Brassica* by the application of colchicine, we observed no effect of the *in vitro* colchicine treatment on embryo formation in the experiments described here (Figure 1) nor in our routine DH program (Additional file 1: Figure S1). We did, however, observe a reduced regeneration rate for the treatments with high colchicine concentrations and extended incubation time (Table 1). A similar trend was observed by Soriano et al. [11] for the wheat cultivar Chris, but not for two other cultivars, indicating that this process is likely genotype dependent.

A critical point for a DH procedure is the survival rate, which gives a measure of how many green plants regenerated on culture medium survive until maturity. We found that this survival rate was only 44% for the *in vivo* approach mainly due to a high mortality after the colchicine treatment of the seedlings. Colchicine is an anti-microtubule agent that not only doubles the chromosomes in the meristem but also has severe effects on the health status of the seedlings, possibly explaining the high mortality [6]. By contrast, the *in vitro* approach resulted in much higher survival rates (Table 1) as the seedlings are not subjected to the deleterious colchicine treatment. In this approach, the survival rate of seedlings appeared to be largely dependent upon the transfer from culture medium to soil.

We found that the rate of spontaneous doubled haploids was 32.1 %, which is consistent with previous reports for triticale [8,9], and confirms that an additional chemically-induced chromosome doubling step is required for triticale DH programs. The *in vitro* colchicine method tested here (Table 2) resulted in improved frequency, with the best treatments more than doubling the rate of fertile plants as compared to the spontaneous doubled haploid rate. More importantly, this method also outperformed the *in vivo* approach. The highest rates of fertile plants were observed for the 1 mM and 5 mM colchicine concentrations applied for 48 or 72 h (Figure 2A). The increasing proportion of fertile plants among the regenerated green plants produced after *in vitro* treatment can be seen in the mosaic plot (Figure 2B). Our results on the number of sterile and fertile tillers per regenerated green plant suggest that the *in vivo* approach mainly produces chimeric plants with sectors that are still haploid (Table 2). By contrast, the plants derived from the *in vitro* approach produced predominantly fertile tillers suggesting that the doubling occurred during an early stage of embryogenesis, and that the plants are entirely doubled haploid. The few sterile tillers may be attributable to inadvertent stress applied to the plants (through transfer to soil or bagging of tillers for example), or to doubling taking place at a later stage of embryogenesis such that a minority of the recovered plants are also mosaic for haploid and doubled haploid sectors.

Whereas the enhancement of the proportion of fertile plants is an important goal, the number of fertile plants derived from a defined number of transferred embryos is the ultimate measure of DH procedure success. In this study we found that this is maximized by three treatment combinations: 1 mM colchicine applied for 24 h, or the lower concentration of 0.3 mM colchicine for 48 or 72 h. The variation in optimal incubation time is advantageous because it gives the experimenter more flexibility, especially in applied high-throughput DH programs. With regard to the rate of fertile plants produced from a standard number of transferred embryos, the *in vivo* approach was only slightly less effective than the *in vitro* approach. This similar rate, however, is misleading since a fair comparison must consider the effort required to reach this value. Culturing of seedlings and fostering of treated plants to maturity, including the bagging required to prevent cross-pollination, are resource and time consuming steps. For the *in vivo* approach, many plants are maintained throughout the entire method but do not yield any DH seeds. By contrast, the higher rate of fertile plants and the higher survival rate observed for the *in vitro* approach more than compensate for the reduced regeneration rate as compared to the *in vivo* approach.

## Conclusion

In conclusion, we have shown that an *in vitro* approach for chromosome doubling performs well for microspore culture of triticale. The higher rate of fertile plants produced per number of transferred embryos and the more economical employment of resources make the *in vitro* approach an attractive alternative to the traditionally used *in vivo* colchicine treatment of seedlings.

## Methods

### Plant material

This study was based on winter triticale (*Triticosecale* Wittmack L.) F_1_ plants derived from a cross between the two cultivars Witon and Corino. Seeds of donor plants were sown in pots in September and kept outside the greenhouse for vernalization until February. Plants were then transferred to the greenhouse with initial temperatures of 12°C for 3 days and subsequently grown at 24°C with a photoperiod length of 16 h.

### Microspore isolation

Microspores were isolated following the general procedure described by Eudes and Amundsen [9]. Tillers from donor plants were harvested when the spikes were approximately 1 cm from emergence from the boot. The spikes were wrapped in foil to maintain humidity and the tillers were stored in flasks containing distilled water in a dark cold room (4°C) for 3 weeks as a cold pretreatment. After this time the tillers were surface sterilized with 70% EtOH and the spikes were extracted under sterile conditions. Spikes with a generally healthy appearance were surface sterilized again and, after removing the awns with scissors, were cut into 2 cm fragments and transferred to a sterile cold 250 ml Waring Blender cup. 100 ml cold mannitol (0.3 M) was added to the blender cup, which was sealed with sterile parafilm, and spikes were blended twice for 7 seconds at 18,000 rpm. The suspension was filtered through sterile mesh (100 μm) into a sterile glass beaker and then transferred to two 50 ml centrifuge tubes. The microspores were pelleted by centrifugation at 100 g for 5 minutes in a cooled centrifuge (4°C), resuspended in 45 ml 0.3 M mannitol and centrifuged again. The pellets were subsequently resuspended in 2 ml 0.3 M mannitol each. The microspore solution was then carefully layered on 5 ml of 21% maltose solution in a 15 ml tube and centrifuged at 100 g for 5 minutes at 4°C. The interphases from both tubes containing viable microspores at the appropriate developmental stage were removed by a Pasteur pipette and transferred to a fresh 15 ml centrifuge tube containing NPB-99 medium. Following a washing step (centrifugation at 100 g for 5 minutes at 4°C) the supernatant was removed and approximately 1 ml NPB-99 medium added to the tube. The microspore concentration was determined using a hemacytometer and the final concentration was set at 33,000 microspores per ml medium.

### Embryo induction and plant regeneration

For embryo induction, microspores were cultured in 35 mm Petri dishes with ~100,000 microspores per Petri dish in 3 ml medium. The induction medium was NPB-99 supplemented with 10 mg/l arabinogalactan-proteins (gum arabic from acacia tree, Sigma-Aldrich G9752) and 10 mg/l arabinogalactan (AG) Larcoll ((+)-Arabinogalactan, Sigma-Aldrich 851361) [17], 200 μg/ml cefotaxime, and 120 g/l Ficoll-400. Three ovaries from sterilized spikes were added to each Petri dish. The dishes were sealed with Parafilm and incubated in the dark at 25°C for 4 weeks.

Regenerated embryos were removed from the Petri dishes and placed on regeneration medium (NPB-99 medium, supplemented with 3.5 g/l gelrite and 1 g/l charcoal) in sterile plastic boxes. Plantlets were regenerated at 20°C with a 16 h light period. Once the plants reached the 2–3 leaf stage they were transplanted to pots with soil and grown under the same conditions. Plants were grown to maturity, several tillers per plant were isolated with bags to prevent cross-pollination and seed set was determined after ripening. The plants with seed set were scored as fertile doubled haploid plants as these determine the success of the procedure. The sterile plants contain haploids, aneuploids, mixoploids, and sterile doubled haploids.

### Colchicine treatment

For the *in vitro* colchicine treatment, isolated microspores (~33,000 per ml; i.e. same concentration as for the embryo induction, thus 3 ml for each embryogenesis Petri dish) were transferred to 15 ml tubes containing induction medium (minus Ficoll to allow for washing and centrifugation) and supplemented with either 0.3 mM, 1 mM, or 5 mM colchicine. The negative control was subjected to the same procedure without colchicine treatment. Three incubation periods were tested: 24 h, 48 h, and 72 h. Three replications were used for each combination of colchicine concentration and incubation time and the presented results are averages over all replicates. The tubes were kept in the dark at 25°C and placed on a shaker to keep the microspores in solution and prevent them from settling. After the incubation time the microspores were pelleted by centrifugation (100 g, 5 minutes), washed once with medium without colchicine, and then transferred to Petri dishes for embryo induction as described above.

As a control for the *in vitro* colchicine treatment, we included an *in vivo* colchicine treatment according to Arzani and Darvey [7]. This step was done with seedlings, which were approximately at the 3-4-leaf stage and which were derived from ten replications (after isolation, microspores were directly placed in the Petri dishes for embryogenesis). Seedlings were submerged with their roots and the meristem in a 0.06% colchicine solution supplemented with 1.5% DMSO and Tween 20 (pH 5.5) for 6 h at room temperature and light. Plants were subsequently rinsed in water for 2 h and then planted in soil. Plants were grown to maturity as described above.

### Statistical analyses

Statistical analyses were conducted using the statistical software R [18]. Mosaic plots were drawn using the vcd package for R. Variance components were determined by the restricted maximum likelihood (REML) method. Significance for variance component estimates was tested by model comparison with likelihood ratio tests in a full random model where the halved P values were used as an approximation [19]. Calculations were performed using the software ASReml 2.0 [20]. Mean comparisons in Tables 1 and 2 were obtained for the binominal traits by calculating confidence intervals with the R package binom (Pearson-Klopper method) or by Fisher’s least significant difference (LSD) tests following ANOVA for the traits with a normal distribution.

## Authors’ contributions

TW designed the study. TW and HPM carried out analyses. TW, MRT and JCR wrote the paper. All authors read and approved the final manuscript.

## Supplementary Material

Additional file 1 **Table S1.** Variance component analysis. **Figure S1.** Effect of *in vitro* chromosome doubling on embryogenesis. Results are shown for three different genotypes (F_1_ crosses) for the classical approach where the microspores are placed in Petri dishes directly after isolation, and for the approach presented here including an *in vitro* colchicine step (1 mM for 24h). Click here for file
