# Supplementary material for: Improved efficiency of doubled haploid generation in hexaploid triticale by in vitro chromosome doubling
Source: BMC Plant Biol. 2012 Jul 18;12:109. doi: 10.1186/1471-2229-12-109 (PMC3443072; doi:10.1186/1471-2229-12-109)
Supplement: Additional file 1 — Table S1. Variance component analysis. Figure S1. Effect of in vitro chromosome doubling on embryogenesis. Results are shown for three different genotypes (F1 crosses) for the classical approach where the microspores are placed in Petri dishes directly after isolation, and for the approach presented here including an in vitro colchicine step (1 mM for 24h). [file 1471-2229-12-109-S1.pdf]

Improved efficiency of doubled haploid generation in hexaploid Triticale by *in vitro* chromosome doubling

Tobias Würschum[1*], Matthew R. Tucker[2], Jochen C. Reif[1], and Hans Peter Maurer[1]

Institutional address:

[1] Tobias Würschum, Jochen C. Reif, Hans Peter Maurer, State Plant Breeding Institute, University of Hohenheim, 70593 Stuttgart, Germany,

[2] ARC Centre of Excellence for Plant Cell Walls, University of Adelaide, Waite Campus, Urrbrae SA 5064, Australia

[*] Corresponding author: Tobias Würschum tobias.wuerschum@uni-hohenheim.de

Table S1 - Variance component analysis

	Regeneration rate	Green Plants	Survival rate	Fertile plants	Fertile plants / 100 embryos
σ^2_{Time}	1.57e-3	2.22e-8	7.09e-3	3.89e-3*	7.43e-9
σ^2_{Conc}	2.37e-8	2.22e-8	2.03e-8	8.32e-3**	3.49e-4*
$\sigma^2_{Time:Conc}$	1.30e-8	4.67e-4	8.40e-3	2.37e-8	7.43e-9

*, ** indicate significance at $P < 0.1$ and $P < 0.05$, respectively

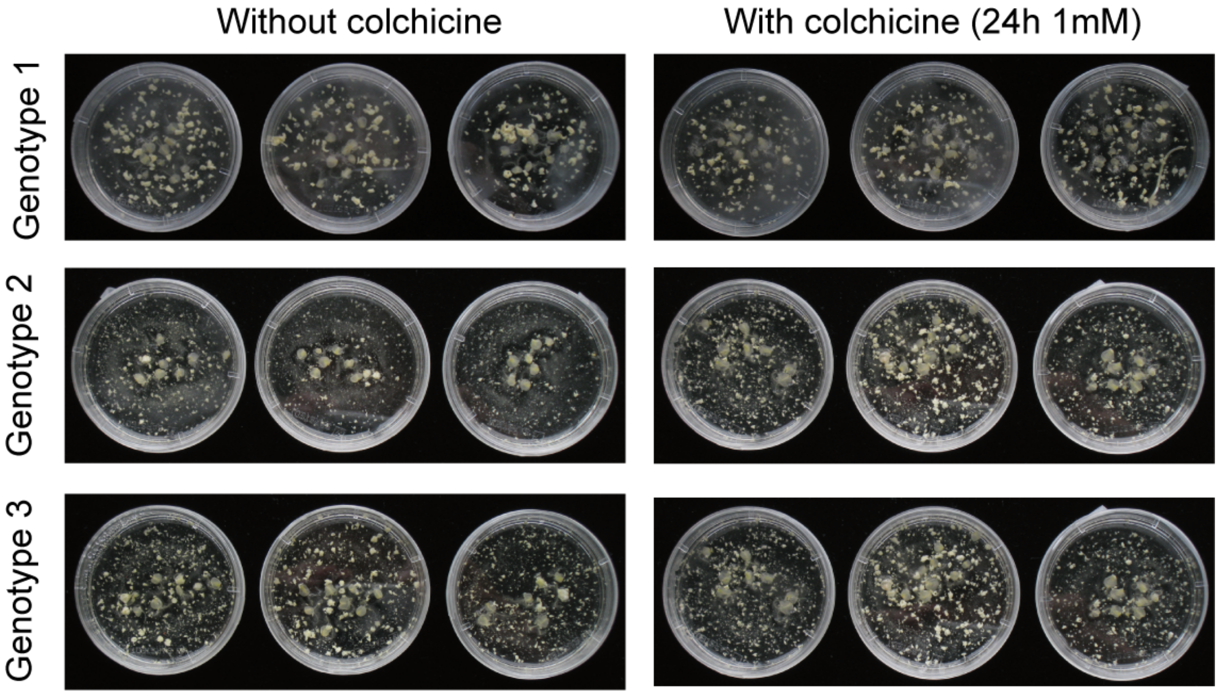

Figure S1 - Effect of *in vitro* chromosome doubling on embryogenesis

Results are shown for three different genotypes (F_1 crosses) for the classical approach where the microspores are placed in Petri dishes directly after isolation, and for the approach presented here including an *in vitro* colchicine step (1 mM for 24h).
